# Perceived potentially inappropriate treatment in the PICU: frequency, contributing factors and the distress it triggers

**DOI:** 10.3389/fped.2024.1272648

**Published:** 2024-01-18

**Authors:** Amrita Sarpal, Michael R. Miller, Claudio M. Martin, Robert W. Sibbald, Kathy N. Speechley

**Affiliations:** ^1^ Department of Paediatrics, Children's Hospital – London Health Sciences Centre, Schulich School of Medicine and Dentistry, Western University, London, ON, Canada; ^2^ Children's Health Research Institute, London, ON, Canada; ^3^ Lawson Health Research Institute, London, ON, Canada; ^4^ Division of Critical Care, Department of Medicine, London Health Sciences Centre, Schulich School of Medicine and Dentistry, Western University, London, ON, Canada; ^5^ Department of Ethics, London Health Sciences Centre, London, ON, Canada; ^6^ Department of Family Medicine, Schulich School of Medicine and Dentistry, Western University, London, ON, Canada; ^7^ Department of Epidemiology and Biostatistics, Schulich School of Medicine and Dentistry, Western University, London, ON, Canada

**Keywords:** death & dying, potentially inappropriate treatment, ethical conflict, medical futility, end-of-life (EOL), distress

## Abstract

**Background:**

Potentially inappropriate treatment in critically ill adults is associated with healthcare provider distress and burnout. Knowledge regarding perceived potentially inappropriate treatment amongst pediatric healthcare providers is limited.

**Objectives:**

Determine the frequency and factors associated with potentially inappropriate treatment in critically ill children as perceived by providers, and describe the factors that providers report contribute to the distress they experience when providing treatment perceived as potentially inappropriate.

**Methods:**

Prospective observational mixed-methods study in a single tertiary level PICU conducted between March 2 and September 14, 2018. Patients 0–17 years inclusive with: (1) ≥1 organ system dysfunction (2) moderate to severe mental and physical disabilities, or (3) baseline dependence on medical technology were enrolled if they remained admitted to the PICU for ≥48 h, and were not medically fit for transfer/discharge. The frequency of perceived potentially inappropriate treatment was stratified into three groups based on degree of consensus (1, 2 or 3 providers) regarding the appropriateness of ongoing active treatment per enrolled patient. Distress was self-reported using a 100-point scale.

**Results:**

Of 374 patients admitted during the study, 133 satisfied the inclusion-exclusion criteria. Eighteen patients (unanimous - 3 patients, 2 providers - 7 patients; single provider - 8 patients) were perceived as receiving potentially inappropriate treatment; unanimous consensus was associated with 100% mortality on 3-month follow up post PICU discharge. Fifty-three percent of providers experienced distress secondary to providing treatment perceived as potentially inappropriate. Qualitative thematic analysis revealed five themes regarding factors associated with provider distress: (1) suffering including a sense of causing harm, (2) conflict, (3) quality of life, (4) resource utilization, and (5) uncertainty.

**Conclusions:**

While treatment perceived as potentially inappropriate was infrequent, provider distress was commonly observed. By identifying specific factor(s) contributing to perceived potentially inappropriate treatment and any associated provider distress, organizations can design, implement and assess targeted interventions.

## Introduction

In 2015, a multi-society (ATS/AACN/ACCP/ESICM/SCCM) policy statement defined potentially inappropriate treatment as treatment sought by a patient or surrogate decision maker that has *some* possibility of achieving a physiological goal (1); however, clinicians believe the treatment is non-beneficial and is “outside the boundaries of accepted practice due to other ethical considerations” (2). Futile treatment was defined as treatment “that (has) no chance of achieving the intended *physiological* goal” (1). Despite establishment of a formal definition, fulfillment of the criteria for potentially inappropriate is a matter of *perception*, and thus like non-beneficial treatment, it includes a subjective values-based judgement (3). The terms, potentially inappropriate treatment and non-beneficial are not synonymous (4). Nonbeneficial treatment emphasizes the lack of expected benefit and the imbalance between the burdens and expected benefits while potentially inappropriate treatment acknowledges that perceptions of appropriateness or benefit are influenced by the preferences, culture and values of patients and providers (4). What constitutes potentially inappropriate is open to interpretation and the associated subjectivity and ambiguity thus prevent objective measurement of potentially inappropriate treatment. To ensure patient goals and values are honored providers in the intensive care unit rely on advance directives and surrogate decision makers to assist in clarifying goals of care based on the patient's known wishes.

As children may not have formed or expressed their own values and wishes due to age or level of cognitive function (5), providers rely heavily on parents and guardians to guide medical decisions. The number of children with chronic critical illness, which is the combination of chronic complex conditions and dependence on medical technology requiring pediatric intensive care unit (PICU) admission, has increased substantially (6, 7). When patients with chronic critical illness require ongoing aggressive treatment, including initiation of invasive medical technology, PICU staff may be more likely to raise concerns about non-beneficial care (3). PICU staff may struggle to meet the needs of these children and their parents and what they believe is in child's best interests; they may *perceive* this treatment as potentially inappropriate and conflict and distress may result (8). Moral distress is one form of distress providers may experience in the PICU (9); it is “the anguish (experienced) in response to a situation in which the person is aware of a moral problem, acknowledges moral responsibility, and makes a moral judgement about the correct action, yet as a result of real or perceived constraints, participates in perceived moral wrongdoing” (10). Previous studies quantified futility and potentially inappropriate treatment based on definitions that are no longer utilized. These studies focused on resource utilization by patients receiving treatment perceived as potentially appropriate (6, 11). In a UK cross-sectional study of PICU patients, the frequency of futility and perceived potentially inappropriate treatment was solely determined by the unit director at a single point in time (12).

The frequency of perceived potentially inappropriate treatment in PICUs and the distress that may be associated has not been examined to our knowledge. Our primary aim was to prospectively quantify the frequency of perceived potentially inappropriate treatment over 6 months from the PICU nurses and physicians' perspective. We also aimed to describe factors associated with perceived inappropriate treatment, identify whether providing treatment perceived as potentially inappropriate is associated with provider distress and explore factors providers suggest contribute to experiencing distress when providing perceived potentially inappropriate treatment.

## Materials and methods

### Study design

We conducted a single-center, prospective, observational mixed-methods study approved by Western University Health Sciences Research Ethics Board (#106981) using a sample of convenience. A waiver of consent was approved for patient participants as no identifiable patient information was collected. Written informed consent was required and obtained for individual providers who chose to participate. Research procedures were conducted in accordance with established local and regional ethical standards, and with the Helsinki Declaration of 1975.

### Hospital setting

The study was conducted in a medical-surgical tertiary level PICU with 800 admissions per year. The multidisciplinary team included bedside nurses, charge nurses, intensivists, a pharmacist, a dietician, a fellow, respiratory therapists (cross-cover several units in the combined adult/pediatric hospital) and residents from a variety of adult and pediatric sub-/specialties who rotated for four weeks in the PICU.

### Patient selection

A research coordinator screened patients in the PICU Monday through Friday between March 2, 2018 and September 14, 2018 for study eligibility. Patients aged 0–17 years inclusive admitted to the PICU for ≥48 h and not medically fit for discharge/transfer at the time of enrollment were included if one of the following was present:

1. Any patient with ≥1 persistent organ system dysfunction
2. Moderate to severe mental and physical disabilities as defined by Baseline Pediatric Cerebral Performance Category (PCPC) (13) score of ≥3 OR baseline Pediatric Overall Performance Category (POPC) (13) score of ≥4 OR baseline Gross Motor Function Classification System (GMFCS) (14) IV or V;
3. Baseline dependence on medical technology including respiratory or feeding support, cerebrospinal fluid shunts, semi-permanent vascular access device or requirement for dialysis.

A patient was excluded from the study if at the time of enrollment, the patient had been (1) admitted to the PICU for less than 48 h, or (2) was medically fit for transfer/discharge to the ward.

### Provider selection

The study was restricted to nursing and attending intensivists who were invited to participate following written informed consent. The entire PICU staff complement included 62 nurses and 6 intensivists.

Patient and provider recruitment were conducted separately.

### Data collection strategy

We collected provider demographic details and assigned each individual a unique code for completion of the Ongoing Active Intervention questionnaire, herein referred to as questionnaire(s). Utilizing previously published results (15), and expert opinion (pediatric palliative care, pediatric intensive care, and health survey experts), we developed an initial questionnaire that was piloted by both PICU and non-PICU staff and refined through an iterative process. The 21-item questionnaire explored: (1) factors contributing to perceived ongoing potentially inappropriate treatment; (2) whether the provider was experiencing distress, and if so, to describe using free text, the factors to which the distress experienced was attributed (Supplementary Material).

On each study day, the bedside nurse of an eligible patient, charge nurse and intensivist on service were asked, “*In your opinion, is ongoing active intervention in the critical care environment for your patient appropriate?*”. The research assistant recorded the provider's answer directly into the research database via a portable electronic device. The survey question was administered privately and individually by a research assistant to ensure individual responses were kept confidential. When ongoing intervention was perceived as appropriate, the research coordinator noted the provider's response and the remainder of the questionnaire was not applicable. When ongoing intervention was *perceived* as *potentially inappropriate (i.e., provider stated “No”)*, the full 21-item questionnaire was administered (See Supplemental Material). The primary objective, frequency of perceived potentially inappropriate treatment, was determined by how often “ongoing active intervention” was considered *inappropriate*, using three levels of agreement (1, 2, or 3 providers), on any given study day. When the research coordinator noted a difference in opinion regarding the appropriateness of ongoing active intervention between the providers, further questions were not asked of providers who believed that ongoing active intervention was appropriate in order to maintain the established survey methodology and to avoid introducing bias and potentially influencing provider responses.

We collected patient demographics and clinical data including Pediatric Risk of Mortality Score III (PRISM III) and pediatric logistic organ dysfunction (PELOD) at enrollment (marked Day 1). We collected provider questionnaire responses and PELOD scores on Days 1, 3, 5 and 7 and weekly thereafter, until patient death or discharge from PICU rather than on consecutive days to avoid questionnaire fatigue. When a study day fell on a weekend, data were collected the following Monday and subsequent study days were deferred to maintain the appropriate gap between data collection times. We collected outcome data, including disposition and survival at 3 months post PICU discharge.

### Statistical Analysis

Continuous variables were summarized using medians and interquartile ranges and comparisons between groups were examined with Mann–Whitney *U*-tests (or Poisson loglinear regression analysis or Spearman's rank correlation coefficient, as appropriate). Categorical variables were summarized using frequencies and percentages, and comparisons between groups were examined with chi-square tests (or Fisher's exact tests, as appropriate). Patients readmitted to the PICU and perceived as receiving potentially inappropriate treatment during more than one admission were considered separate cases when analyzing patient demographics; however, given level of agreement between providers for these patients did not change over time, these patients were included only once when determining the frequency of perceived potentially inappropriate treatment. All analyses were conducted using SPSS v24 (IBM Corp., Armonk, NY, USA).

Qualitative content analysis of free text responses was conducted after closure of the questionnaire portion of the study using NVivo 12 Pro to manage and code verbatim abstractions and identify “nodes”. A detailed review of the nodes, sub-nodes and related text was independently reviewed by two coders (SC and AS) to confirm the categorization and to consolidate overlapping nodes where possible to establish subthemes. Coders subsequently met and through consensus achieved the first stage of thematic categorization. Subsequent analysis, was performed by a single coder (AS) to further consolidate subthemes into overarching themes.

## Results

### Potentially inappropriate treatment patient data

**Figure 1:**
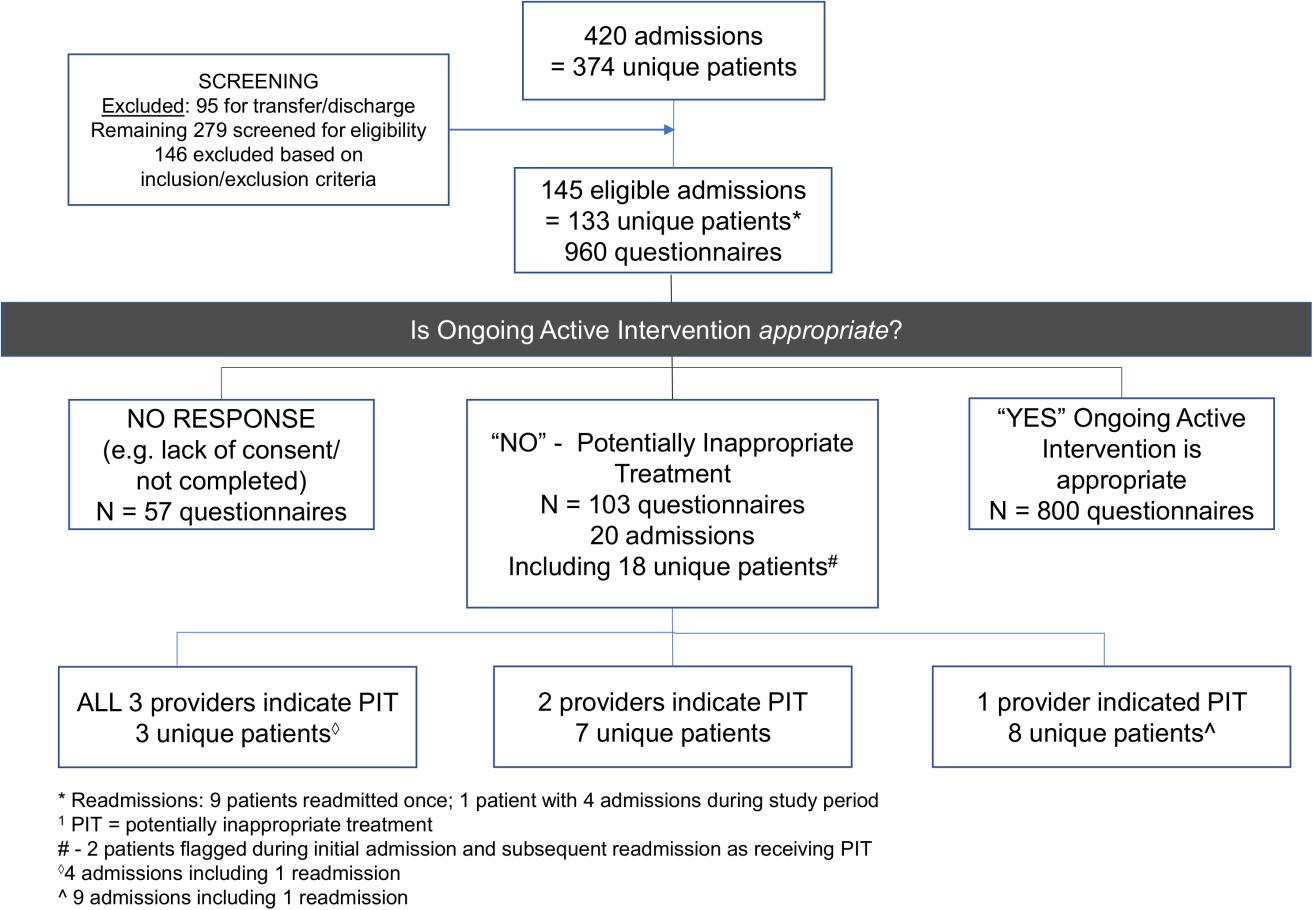
Patient flow chart, study questionnaire completion and frequency of potentially inappropriate treatment as perceived by health care providers.

During the enrollment period, 420 PICU admissions among 374 unique patients were documented; 133 unique patients met the inclusion criteria (Figure 1). Three participating providers (concurrent bedside nurse, charge nurse and intensivist on duty) unanimously perceived treatment to be potentially inappropriate in 3/133 (2%) patients. One patient had a chronic complex condition, limited cognitive function and a need for medical technology; while another developed the aforementioned following severe hypoxic ischemic injury due to prolonged out of hospital cardiac arrest. The third had advanced cancer with an extremely poor prognosis. Two of the three providers concurrently perceived treatment to be potentially inappropriate in 7/133 (5%) patients and a single provider indicated they perceived potentially inappropriate treatment in 8/133 (6%) patients. The mortality rate at 3-months post PICU discharge was 3/3 (100%), 4/7 (57%) and 0/8 (0%) for the three groups, respectively. Thus, the mortality rate for patients receiving potentially inappropriate treatment as perceived by one or more providers was 7/18 (39%), while the overall PICU mortality rate in 2018 was 22/782 (2.8%) with (*p* < 0.001). Of the 18/133 patients (14%) perceived as receiving potentially inappropriate treatment, 2 patients required PICU readmission during the study period. Perceived potentially inappropriate treatment was associated with a higher PRISM score (*p* = 0.02), higher PELOD score (*p* = 0.03), and longer PICU length of stay (LOS; *p* < 0.001) but shorter overall hospital LOS (*p* < 0.001) Table 1.

**Table 1:** Patient demographics.

Variable^a^	Perceived as receiving potentially inappropriate treatment (*N* = 20)^b^	Not potentially inappropriate treatment (*N* = 121)^c^	*P*-value
Age			
Neonate (0–<1 month)	2 (10)	12 (10)	*P* = 0.615
Infant (1–12 months)	3 (15)	24 (20)	
Child (>1–12 years)	8 (40)	59 (49)	
Adolescent (>12–17 years)	7 (35)	26 (21)	
Male	11 (55)	63 (52)	*P* = 0.808
PICU LOS, days	6.5 (3.75–12.50)	4.0 (2.00–7.00)	*p* < 0.001
Hospital LOS, days	6.5 (5.25–83.25)	10.00 (5.00–23.00)	*p* < 0.001
Prism score, initial	8.5 (0.25–18.00)	3.00 (0–5.00)	*p* = 0.02
Pelod score, initial	7.0 (5.00–10.00)	5.00 (3.00–7.00)	*p* = 0.03
Patient condition			
Acute condition	5 (25)		
Chronic complex condition			
With severe physical/mental disabilities and/or baseline medical technology	11 (55)		
Without severe disabilities or baseline medical technology	4 (20)		

^a^ Continuous variable as median (IQR); categorical variables as *n* (%).

^b^ Includes 2 readmissions.

^c^ Includes 6 readmissions.

### Provider data

The total PICU staff complement consists of 68 providers (62 nurses and 6 physicians). Sixty-one of 68 eligible providers (56/62, 90% nurses; 5/6, 83% physicians) consented to participate in the study, and 54/61 (88.5%) provided demographic details (Table 2). Questionnaires not completed due to non-participation were marked “missing” for an overall questionnaire completion rate of 94%. Each provider was surveyed a median of 7.5 times (IQR 4.25–12.75) in 6 months. Thirty-three individual providers stated “ongoing active intervention” was not appropriate for the patient 103 times and subsequently completed the 21-item questionnaire. An individual provider completed the full 21-item questionnaire a median of 2 times (IQR 1–4).

**Table 2:** Provider demographics.

Variable	*n* (%)
Provider type^a^	
Physician	5 (9.3)
Nurse	49 (90.8)
Age (years)	
18–25	15 (27.8)
26–35	15 (27.8)
36–45	9 (16.7)
46–55	9 (16.7)
56–65	6 (11.1)
Years in practice	
0–2	13 (24.1)
3–5	11 (20.4)
6–10	9 (16.7)
11–20	7 (13.0)
>20	14 (25.9)
Years in PICU	
0–2	19 (35.2)
3–5	7 (13.0)
6–10	9 (16.7)
11–20	8 (14.8)
>20	11 (20.4)
Employment status	
Casual	1 (2.1)
Part time	13 (27.1
Full time	34 (70.8)
Marital status	
Single	16 (29.6)
Married	25 (46.3)
Common-law	10 (18.5)
Divorced/separated	3 (5.6)
Religious affiliation	
Christian	31 (57.4)
Hindu	2 (3.7)
Nonreligious	18 (33.3)
Other	3 (5.6)

^a^ PICU staff composition: 6 physicians; 62 nurses. Participating providers: 61; data is missing for 7 participating providers.

The most common reason that providers indicated for perceiving treatment as potentially inappropriate was extremely poor current quality of life; the most common solution providers cited to resolving the situation of perceived potentially inappropriate treatment was patient discharge or demise. A surrogate decision maker's request to continue treatment was the most common reason for ongoing active intervention. In 8/18 (44%) of the patients perceived as receiving potentially inappropriate treatment, one or more providers reported not having a good sense of the patient's/family's wishes, goals and values. While very important, verifying documentation summarizing the patient's/family's goals (if known) was not included within the scope of the current study (Table 3).

**Table 3:** Reasons providers cited for why ongoing active intervention was ongoing and was potentially inappropriate, their degree of understanding of patient/family wishes and potential solutions they cited to resolve the situation.

Reasons why treatment is potentially inappropriate cited	Number of surveys *n* = 103 (%)^a^
The patient's current quality of life is extremely poor	80 (77.7)
The burden of treatment outweighs the benefit	70 (65.0)
The patient will most likely not survive outside of the PICU	62 (60.2)
Patient lacks capacity to appreciate the benefit of ongoing active intervention	59 (57.3)
Other patients could benefit more from the resources being provided to this patient	46 (44.7)
Death is imminent with no reasonable chance of survival	45 (43.7)
Ongoing treatment is inconsistent with the patient's (if known) or the substitute decision maker's goals	1 (1)
Other (Free text) • No policy/procedure exists to hold parents accountable for their demands and choice • Known poor prognosis	2 (2)
Reason why treatment is ongoing	Number of surveys *n* = 166^b^
SDM wants to continue ongoing active intervention	80 (48.2)
Issue is being addressed but needs more time (requires testing and/or family meeting)	31 (18.7)
Clinicians would like to avoid conflict or legal confrontation with SDM	20 (12)
Understanding of patient/family's goals, wishes and values	Number of surveys *n* = 98 (%)^c^
I have a good sense of … from the patient	4
I have a good sense of … from written documentation previously completed by patient/family	26
I have a good sense of … from discussions with the family	45
I do not have a good sense of the patient's/family's wishes	23
Solution cited	Number of surveys *n* = 97 (%)^d^
Patient death or discharge	36 (37%)
Legal intervention seeking a change in treatment plan	28 (29%)
Initiating/repeating discussions with SDM to discuss limiting active treatment including withdrawal of life sustaining treatment	25 (26%)
Team should unilaterally refuse to provide ongoing active intervention including life sustaining treatment	3 (3%)
Team should wait until the SDM initiates discussions about ongoing active intervention	3 (3%)
Team should seek an ethics consultation	2 (2%)

PICU, pediatric intensive care unit; SDM, surrogate decision maker.

^a^ Multi-select question. Total number of reasons (*n* = 366) selected on 103 completed questionnaires.

^b^ Multi-select question. Total number of reasons (*n *= 166) selected on 103 completed questionnaires. Three most common reasons given are shown.

^c^ Single-select question. Providers answered this question 98/103 questionnaires; 5 did not answer.

^d^ Single-select question. Providers answered this question 97/103 questionnaires; 6 did not answer; 9 answered “other” and provided free text answer. For 9/9 free text answers, the answer could be assigned to a listed category.

### Provider distress

Providers reported experiencing distress secondary to providing treatment they perceived as potentially inappropriate in 93/103 (90%) questionnaires. Thirty-three providers (5/5, 100% participating physicians; 28/56, 50% participating nurses) reported experiencing distress at least once (median = 2, IQR = 1–4) with a median (IQR) intensity of distress measuring 65 (55–81) out of 100 during the study. Seventy-five percent (70/93) of questionnaires in which distress was reported were linked to 3 unique patients (1 required readmission). No significant relationship was found between provider type and the reported intensity or frequency of distress (*p* = 0.15). No significant correlation was found between frequency and average intensity of distress experienced by an individual provider (*p* = 0.24).

Initial coding of provider statements regarding factors to which they attributed their distress revealed more than 10 potential subthemes. Secondary analysis aimed at consolidating and collapsing the subthemes produced five overarching themes: (1) suffering including a sense of causing harm, (2) conflict, (3) quality of life, (4) resource utilization, and (5) uncertainty. Suffering was experienced as (a) existential or physical, (b) of the patient, patient's family, or others, and (c) of the provider through a sense of inflicting harm through continued ongoing treatment (Table 4). Conflict was experienced overtly through communication with families described as combative and argumentative, and through openly expressed distrust of the medical team. It was also experienced passively through lack of parental engagement and absent or challenging communication. Although infrequent (*n* = 4 responses), conflict within the medical team also contributed to provider distress. With prolonged ongoing treatment that was perceived as potentially inappropriate, two separate providers reported “desensitization” and cited it as a protective factor against experiencing further distress.

**Table 4:** Initial coding framework, including example text, and overarching themes providers attributed their distress to when providing potentially inappropriate treatment.

Final themes	Number of participants (%)			Initial coding framework	Example text^a^
	Nurse (*n* = 27)	Physician (*n* = 5)	Total (*n* = 32)		
Suffering and sense of causing suffering	17 (63)	5 (100)	22 (69)	Prolongation of death Questionable benefit of treatment Sense of causing harm Treatment burden Desensitization Indignity Suffering of others Suffering of the child (existential, physical)	“My distress stems from the fact that we have all played a part in robbing this child of her dignity; we are denying her the chance to have a peaceful death”. “It is difficult to watch a patient suffer when outcome is poor-to-none and quality of life is poor with little-to-no improvement…” “Having to complete routine care/orders/etc. that cause patient +++ discomfort or pain. (I) feel that patient's wishes may differ from what (the) parent(s) are wanting, not sure patient would want to go through this”. “I'm experiencing an internal ethical dilemma because I feel like the patient has a poor quality of life and our treatment is doing more harm than good”. “the unfairness to the p(atien)t. As we watch her slowly dying… (b)eing unable to use human kindness”
Conflict	13 (48)	5 (100)	18 (56)	Conflict due to family directed care Conflict family vs. provider Conflict between providers Perceived incongruence of child vs. parents’ wishes Lack of parental understanding Difficult communication	“(T)hey are delaying the difficult decision to withdraw life sustaining treatment. They understand (the) prognosis but can't face the decision. We do not want to … destroy the tenuous therapeutic relationship. (The) family has a lot of negative feelings about the health care team, which makes conversations with them very difficult”. “I feel for the mother who does not want to feel guilty for withdrawal and whose family members tell her withdrawal is killing the baby even though they are too distressed by the sight of the (endotracheal tube) to visit. I am frustrated and sad for the baby and not too distressed because I am desensitized, empathize with the mother and know death is imminent”
Quality of life	14 (52)	2 (40)	16 (50)	Poor quality of life Quality of life	“It is difficult to watch a patient suffer when outcome is poor-to-none and quality of life is poor with little-to-no improvement…”
Resource utilization	7 (26)	2 (40)	9 (28)	Resource allocation Wasted resources/dollars Societal burden	“It is extremely frustrating to see health care dollars being wasted on this patient. … we had to turn away other viable patients due to this patient occupying a PICU bed”. “While she has been admitted, patients requiring admission for ICU treatment have been declined/transferred elsewhere”.
Uncertainty	8 (30)	1 (20)	9 (28)	Uncertainty about direction of care Uncertainty what the child wants vs. parental wishes Uncertainty regarding perceptions Lack of consistency Uncertainty regards to duration Uncertainty of understanding	“Frustration with lack of direction with plan of care”. “(I have an) incomplete understanding of the parents’ wishes for the patient; if their wishes are in line with the patient's best interests”. “significance of recent deterioration may not be understood by parents as they have not been at the bedside and I have not been able to update them yet”.

^a^ Initial coding of free text responses identified multiple ideas/themes.

## Discussion

The overall frequency of perceived potentially inappropriate treatment in the current study approached 14% based on three differing levels of agreement among providers. Greater degree of provider consensus was associated with higher mortality on follow-up 3 months post PICU discharge. Most importantly, more than one-half of providers experienced distress secondary to providing treatment they perceived as potentially inappropriate.

In a 1996 prospective cohort study including 353 children in an American PICU, 6.5% patients met the definition of futility (6): (1) Imminent demise futility (PRISM score with high risk of mortality); (2) lethal condition futility (long-term survival unlikely); or (3) qualitative futility (high morbidity). Using similar definitions, Goh and Mok reported a 5.1% futility rate in a UK PICU (11), while a cross-sectional point prevalence survey of UK PICU directors suggested 21% of all care was perceived as potentially inappropriate (13%) or futile (8%) (12). The definitions utilized in these studies differ from the current SCCM definitions; additionally, prior to 2015, the terms futility and potentially inappropriate treatment were often used interchangeably. In addition, the number of children with chronic complex conditions and chronic critical illness requiring PICU has increased substantially. Sachdeva et al. noted 89% of their children had no developmental delays; they did not specifically comment on how many of the remaining children had complex chronic conditions or were dependent on medical devices (6). In comparison, in 2010, Burns et al. reported 50% of children requiring PICU admission had a chronic complex condition (7). Variability in definitions, changes in patient demographics including the presence of chronic conditions, dependence on medical technology and differences in study methodology may explain the differing rates of perceived potentially inappropriate treatment observed. In addition, whether treatment is perceived as potentially inappropriate is a matter of *opinion* and thus subject to variability (4).

In cases of perceived potentially inappropriate treatment, conflict may arise due to communication issues and lack of understanding of patient/family's wishes. The importance of communication including multidisciplinary case conferences was evident based on provider responses utilizing the study questionnaire and the variable opinions regarding who was perceived as receiving potentially inappropriate treatment. Case conferences are an important means to develop rapport and trust, and establish goals of care through shared decision making (16). In a practice innovation, Wocial et al. (17) introduced weekly Pediatric Ethics and Communication Excellence (PEACE) rounds aimed at establishing realistic goals of care for PICU patients. In the pre-post intervention analysis, a statistically significant reduction in PRISM indexed length of stay, increase in a change in code status to do not resuscitate and increase in-hospital death while no change in patient 30- and 365-day mortality (17) was observed. While early and proactive communication is encouraged (1, 2) and effective (17) due to time constraints and competing demands, PICU providers may avoid case conferences thereby perpetuating the status quo and what may be perceived as potentially inappropriate treatment (18). Delays in initiating end-of-life discussions, indecision regarding perceived potentially inappropriate treatment and lack of nursing participation in case conferences (19) may contribute to distress and lead to conflict within the team. Many of our nurses did not have a good understanding of the patient's/family's wishes. Unfortunately, patient/family wishes are frequently not discussed (20), or may be poorly documented (21). Improving communication via regular multidisciplinary case discussions (17, 22), early consultation with experts in clinical ethics and palliative care (1, 2), clear documentation of patient/family wishes and goals of care, and team debriefing may reduce conflict (23), provider distress and the frequency of perceived potentially inappropriate treatment.

Half of our providers reported distress, which was primarily centered around 3 patients who required an extended PICU stay and had or developed a chronic critical illness or had an extremely poor prognosis. The PICU environment, staffing model and training programs were not developed with the needs of children with chronic critical illness in mind (3). Rapid staff turnover, gaps in continuity of care and provider discomfort in managing patients with chronic critical illness contribute to conflict (24), distress and perceived potentially inappropriate treatment. These children have a higher mortality rate (7), and use a disproportionate amount of healthcare resources compared to other PICU patients (25), leading providers to question the appropriateness of continued treatment. Providers noted their distress stemmed from observing ubiquitous suffering (patient, family, self and colleagues), conflict with family and each other, compromised quality of life, and uncertainty (e.g., direction of care). Miles et al. noted similar themes noting treatment perceived as “non-beneficial” was the most common source of provider distress (3). In a recent multicenter study, Dryden-Palmer et al. noted moral distress was common among pediatric and neonatal ICU providers (26). High scoring questions were linked to initiating and continuing life support the provider did not agree with, offers of “false hope”, and quality of care suffering due to non-continuity (26). Importantly, provider predictions regarding patient survival and functional outcome tend to pessimistic and in relation to children with medically complex conditions, inaccurate (27); providers frequently underestimate patients' quality of life (28). By proactively engaging families in regular discussions regarding the patient's condition, realistic and appropriate goals of care including resuscitation status, can be established (17). It has been postulated that witnessing prolonged potentially inappropriate treatment may lead to more intense distress or desensitization (29); interestingly, we observed the latter. Perceived potentially inappropriate treatment is independently associated with burnout, intention to quit (30, 31) and provider distress. In cases of perceived potentially inappropriate treatment, we suggest clinical staff be supported and empowered to find evidence-based solutions that consider individual and unit needs and local culture.

Strengths of the current study include its prospective and longitudinal design, and use of an interviewer-administered questionnaire with a (94%) response rate exceeding those seen in comparable adult studies (15). As a single center study, our results may reflect local unit issues rather than issues universal to PICU. However, similar themes have been identified by others lending merit to our findings. Other limitations include missing data, lack of daily surveys and not including the full multi-disciplinary team, which may have impacted study outcomes. To avoid introducing bias, follow up questions were not posed when providers differed in their opinion regarding the appropriateness of ongoing treatment; this may have resulted in the loss of potentially important data. As well, the families' voices were not included in the current study as, and as noted by Lo, families' may differ in regard to their definition of potentially inappropriate and/or non-beneficial treatment (4). Furthermore, providers may have opted to state treatment was appropriate to avoid completing the 21-item questionnaire thereby underestimating the true frequency of perceived potentially inappropriate treatment.

## Conclusion

While perceived potentially inappropriate treatment may be infrequent, distress secondary to treatment perceived as potentially inappropriate is common. Interventions to address provider distress should be developed using established frameworks. Targeted interventions including: (1) strategies to improve communication with families and amongst providers, (2) clear and concise end-of-life treatment recommendations, and (3) advocacy for a congruent care plan that could alleviate conflict and uncertainty, may decrease instances of perceived potentially inappropriate treatment, and may decrease distress associated with providing perceived potentially inappropriate treatment. Future areas for research include: (1) quantification of perceived potentially inappropriate treatment and moral distress in other contexts and perhaps on a national or international scale (including the full multidisciplinary team and learners), (2) Exploring patient and families' perspectives with regards to perceived potentially inappropriate treatment, and (3) implementing strategies to reduce provider distress and perceived potentially inappropriate treatment.

## Data Availability

The raw data supporting the conclusions of this article will be made available by the authors, without undue reservation.
