# Supplementary material for: Perceived potentially inappropriate treatment in the PICU: frequency, contributing factors and the distress it triggers
Source: Front Pediatr. 2024 Jan 18;12:1272648. doi: 10.3389/fped.2024.1272648 (PMC10830678; doi:10.3389/fped.2024.1272648)
Supplement: Supplementary file 1 [file Datasheet1.docx]

**Supplement: Study Questionnaire**

1. In your opinion, is **ongoing active intervention** (OAI) in the critical care environment for your patient **appropriate**?

🞎 YES 🡪 STOP

🞎 NO

2. Why do **you** feel that OAI is **inappropriate** (circle **all applicable** letters)?

1. Death is imminent with no reasonable chance of survival
2. The patient will most likely not survive outside of PCCU
3. Ongoing treatment is inconsistent with the patient’s (if known) or the substitute decision maker’s goals
4. In your opinion, the burden of treatment outweighs the benefit
5. Other patients could benefit more from the resources being provided to this patient
6. In your opinion, the patient’s current quality of life is extremely poor
7. Patient lacks the capacity to appreciate the benefit of OAI
8. Other: _______________________________________________________________

3. To the best of your knowledge is the patient aware of his/her condition and/or prognosis?

🞎 NO

4. What is the **most important** reason for why you think that is?

1. Too young to have developed understanding and capacity
2. Suffers from Global Developmental Delay and has not developed nor is likely to develop capacity/understanding
3. Patient has not been informed
4. New onset neurological injury and is no longer capable
5. Intubated/ventilated, sedated and thus unable to communicate
6. Other: ______________________________________________________________

🞎 YES

5. Do you believe your patient wants the treatment he/she is receiving (circle letter)?

1. YES
2. NO
3. I do not know

6. Are medical decisions being made by a substitute decision maker (i.e. not the patient)?

🞎 NO 🡪 Go to # 10

🞎 YES

7. The substitute decision maker is

1. Parent
2. Family member other than a parent
3. Children’s Aid Society (CAS)
4. Other: ________________________________________________________________

8. To the best of your knowledge does the substitute decision maker understand the condition and

prognosis the patient is facing?

🞎 NO

9. Why is that? (Circle **ALL** that apply)

1. Primary team has not informed substitute decision maker regarding the nature of the underlying condition
2. PCCU team has not informed substitute decision maker of the seriousness of the current clinical picture
3. Substitute decision maker does not agree with the PCCU team’s opinion on the seriousness of the clinical picture
4. Substitute decision maker appears unable to appreciate the seriousness despite being informed by the PCCU team
5. Substitute decision maker appears unable to fully process the information given their current level of stress
6. Underlying diagnosis has yet to be identified
7. Prognosis unknown or uncertain
8. Other: _________________________________________________________

🞎 YES

10. Why do you think treatment is ongoing? (Circle **ALL** appropriate answers; rank your top 3 reasons: 1= most important, 2 = second most important reason, etc.)

1. Are waiting for family to arrive
2. Patient wants to continue OAI
3. Substitute decision maker wants to continue OAI
4. Clinician(s) wants to continue OAI as they feel it is in the patients best interest
5. Clinician(s) would like to avoid conflict or legal confrontation with substitute decision maker /patient
6. Lack of communication between the treating team and the patient/ substitute decision maker
7. Members of the treating team disagree about ideal plan of treatment
8. A non ICU medical/surgical/consulting team wants to continue OAI
9. The issue is being addressed (requires testing and/or family meeting) but needs more time
10. This issue has not been addressed
11. There is a degree of uncertainty regarding the patient’s prognosis/outcome.
12. Other (please explain) ________________________________________________________________________

11. Since you last completed the questionnaire, has the PCCU team and either the patient or substitute decision maker had formal discussions about the patient’s wishes and goals of care/treatment?

🞎 NO

🞎 YES

🞎 I don’t know

12. Describe your understanding of the patient’s/family’s goals/wishes/values (circle **ONE** letter)

1. I have a good sense of the patient's/family’s wishes, values and goals from the patient
2. I have a good sense of the patient's/family’s wishes, values and goals from written documentation previously completed by the patient/family/substitute decision maker
3. I have a good sense of the patient's/family’s wishes, values and goals based on discussions with the family
4. I do not have a good sense of the patient's/family’s wishes

13. Do you believe the family’s expressed wishes/values and goals are in alignment with the child’s best interests?

🞎 NO

🞎 YES

14. In the last week, has the PCCU team discussed the appropriateness of OAIs?

🞎 NO

🞎 YES

15. In the last week, have you been able to express your opinion about the appropriateness of OAIs for this patient to the PCCU team?

🞎 NO

🞎 YES

16. Is there a **communication** issue leading to ongoing active intervention(s) in the critical care unit of this patient?

🞎 NO

17. Is there an **education** issue that is leading to OAI of this patient?

🞎 NO – go to #20

🞎 YES

18. Which of the following is the most important contributor to the knowledge issue (Circle the **ONE** answer you think is the biggest reason even if there may be multiple reasons)?

1. Poor patient understanding of the benefits/burdens of treatment
2. Poor substitute decision maker understanding of the benefits/burdens of treatment
3. Poor understanding of the benefits/burdens of treatment by a member of the treating team
4. Based on the literature, there is uncertainty regarding outcomes/benefits/burden of treatment for this particular patient

🞎 YES

19. Which of the following is the most important contributor to the communication issue? (Circle the **ONE** answer you think is the biggest reason even if there may be multiple reasons)

1. Inability of treating team to agree/reconcile differences in approach to medical treatment/care with the patient/ substitute decision maker because of cultural/religious differences
2. Inability of treating team to communicate with the patient/substitute decision maker because of language differences
3. Inability of treating team to communicate with the patient/substitute decision maker for other reasons (ie. Not due to culture, religion or language issues)
4. Inability of treating team members to communicate with each other
5. Poor patient/substitute decision maker understanding of the benefits/burdens of treatment
6. Poor understanding by the treating team member of the benefits/burdens of treatment
7. Substitute decision maker does not agree with or is unable to process information shared by the PCCU team in regards to the condition/prognosis
8. Medico-legal considerations
9. The benefit/burden of OAI is unclear
10. Other: __________________________________________________________

20. The following scale relates to the degree of distress you may be experiencing as a result of the ongoing treatment of this patient. Please circle the number corresponding to where you feel you are on the continuum. (Scale to be shown to participant)

0 1 2 3 4 5 6 7 8 9 10

Not at all Extremely

Distressed Distressed

21. For those participants selecting 1 or higher on the scale, please ask them to describe what they feel is leading/contributing to their level of experienced distress?

________________________________________________________________________________________________________________________________________________________________________________________________________________________________________________________________________________________________________________________________________________________________________________________________________________________________________________________________________________________________________________________________________________________________________________________________________________________________________________________________________________________________________________________________________________________________________________________________________________________________________________________________________________________________________________________________________________________

22. In your opinion, of the following, which **ONE** expresses the best means of resolving this case of inappropriate OAI for your patient? (Circle the **ONE** answer you think is the best means even if there may be multiple reasons)?

1. There will be no resolution until death or discharge occurs
2. The treating team should ask for a legal intervention to change the treatment plan
3. The treating team should unilaterally refuse to provide ongoing active treatment including life sustaining treatment
4. The treating team should wait until the patient/ substitute decision maker initiates a discussion about ongoing active interventions/life sustaining treatment
5. The treating team should initiate and repeat discussions with the patient/ substitute decision maker to discuss limiting active treatment/withdrawal of life sustaining treatment
6. The treating team should obtain an ethics consultation
7. Other ____________________________________________________________________________________
